# Monitoring of breathing motion in image-guided PBS proton therapy: comparative analysis of optical and electromagnetic technologies

**DOI:** 10.1186/s13014-017-0797-9

**Published:** 2017-03-31

**Authors:** Giovanni Fattori, Sairos Safai, Pablo Fernández Carmona, Marta Peroni, Rosalind Perrin, Damien Charles Weber, Antony John Lomax

**Affiliations:** 1 Center for Proton Therapy, Paul Scherrer Institut, 5232 Villigen, PSI Switzerland; 2 Radiation Oncology Department, Inselspital Universitätsspital Bern, 3010 Bern, Switzerland; 3 Department of Physics, ETH-Hönggerberg, 8093 Zurich, Switzerland

**Keywords:** Respiratory motion, Optical tracking, Electromagnetic tracking, Proton therapy, Gantry, CT imaging

## Abstract

**Background:**

Motion monitoring is essential when treating non-static tumours with pencil beam scanned protons. 4D medical imaging typically relies on the detected body surface displacement, considered as a surrogate of the patient's anatomical changes, a concept similarly applied by most motion mitigation techniques. In this study, we investigate benefits and pitfalls of optical and electromagnetic tracking, key technologies for non-invasive surface motion monitoring, in the specific environment of image-guided, gantry-based proton therapy.

**Methods:**

Polaris SPECTRA optical tracking system and the Aurora V3 electromagnetic tracking system from Northern Digital Inc. (NDI, Waterloo, CA) have been compared both technically, by measuring tracking errors and system latencies under laboratory conditions, and clinically, by assessing their practicalities and sensitivities when used with imaging devices and PBS treatment gantries. Additionally, we investigated the impact of using different surrogate signals, from different systems, on the reconstructed 4D CT images.

**Results:**

Even though in controlled laboratory conditions both technologies allow for the localization of static fiducials with sub-millimetre jitter and low latency (31.6 ± 1 msec worst case), significant dynamic and environmental distortions limit the potential of the electromagnetic approach in a clinical setting. The measurement error in case of close proximity to a CT scanner is up to 10.5 mm and precludes its use for the monitoring of respiratory motion during 4DCT acquisitions. Similarly, the motion of the treatment gantry distorts up to 22 mm the tracking result.

**Conclusions:**

Despite the line of sight requirement, the optical solution offers the best potential, being the most robust against environmental factors and providing the highest spatial accuracy. The significant difference in the temporal location of the reconstructed phase points is used to speculate on the need to apply the same monitoring system for imaging and treatment to ensure the consistency of detected phases.

## Background

Proton therapy based on pencil beam scanning (PBS) exploits the favourable spatial dose distribution of proton beams for the delivery of highly conformal radiotherapy [1]. However, the physiological deformation of anatomical structures, due to respiratory organ motion, can interplay with the dynamics of dose delivery and, if not taken into account, can detrimentally affect treatment quality for mobile tumours in the thorax and abdomen [2]. A key aspect for effective treatment of such tumours therefore, is an accurate assessment of respiration-induced anatomical changes occurring prior to, and during, irradiation [3].

Image guided radiotherapy for the treatment of mobile tumours requires combining 4DCT information, used to model patient motion during treatment planning, with on-line monitoring of motion during treatment. Even though prospective reconstruction methods have been proposed [4, 5], retrospective sorting of 4DCT images is still primarily based on the synchronized acquisition of a breathing surrogate signal during imaging [6]. However, during treatment delivery, external respiratory surrogates are often preferred to X-ray imaging, in order to limit the amount of additional non-therapeutic dose for the patient. As such, optical surface imaging and electromagnetic tracking of implanted transponders are increasingly popular solutions for motion monitoring in radiotherapy, despite the risks of the latter approach of clinical complications [7, 8], artefacts in CT and MR imaging [9, 10] or detrimental effects on range and dose calculations for particle therapy [11–13].

For PBS proton therapy, the size of the gantry, together with the need to bring the treatment nozzle close to the patient to minimize pencil beam sizes [14, 15], provide however additional challenges for motion mitigation over and above those for conventional therapy, for instance due to possible ‘line-of-sight’ problems for optical systems or potential environmental distortions for electromagnetic solutions. The choice of a specific motion monitoring solution for PBS proton therapy is, therefore, strongly influenced by the working environment and treatment unit design. Additionally, a growing number of proton therapy facilities have installed in-room CT [16], which can be used for daily three-dimensional imaging [17, 18], but also to verify, immediately before treatment, the correlation between internal motions and the acquired motion surrogate [19, 20]. Due to the large footprint of such scanners however, imaging is necessarily performed a few meters away from the treatment isocenter, and has to rely on robotic positioning systems for automated patient transportation and setup correction, requiring sophisticated calibration procedures for coordinate transfer between the systems [21]. In such an environment, it is advantageous that any motion monitoring equipment is mounted on the couch, such that it moves together with the patient between imaging and treatment devices. This facilitates the transfer of motion information from imaging to treatment with minor impact on the clinical procedure. It is the aim of this study therefore to compare the practicalities and precisions of table mounted optical and electromagnetic technologies for surface motion monitoring of PBS proton therapy treatments. For this, two representative, and cost effective systems, one of each type and from the same manufacturer, have been comprehensively tested. Real time functionality, and the ease of setting them up on the treatment couch have driven the selection process, thus ruling out surface-based methods like VisionRT (VisionRT Ltd. UK), free standing cameras systems such as BTS SMART (BTS Bioengineering, Garbagnate, IT) solutions [22], or the Varian Calypso**®** (Varian Medical Systems Inc., Salt Lake City, US) wireless electromagnetic sensor.

## Methods

Two motion monitoring technologies for medical applications, both produced by Northern Digital Inc. (NDI, Waterloo, CA), have been compared in this work - the Polaris SPECTRA optical tracking system (OTS) and the Aurora V3 electromagnetic tracking system (EMTS).

Polaris SPECTRA is a position sensor that measures the location of either active or passive infrared markers. Three-dimensional localisation relies on stereo photogrammetry theory to triangulate marker points from multiple calibrated views of the scene that are acquired with two sensors embedded in the device. The NDI Aurora system found instead its tracking principle on electromagnetic field. The measurement is based on two key elements, a field generator used to create a varying magnetic flux and coil sensors. Induced coil voltage is readout to determine the sensor spatial location based on the prior knowledge of strength and phase of the exciting field.

OTS and EMTS have been compared both technically, by measuring tracking errors and system latencies under laboratory conditions, and clinically, by assessing their practicalities and sensitivities when used with imaging devices and PBS treatment gantries. Our study is limited to couch mounted solutions that represent the simplest option for continuous patient monitoring during the entire treatment process. For all tests, the sampling rates for the two systems were set to the maximum allowed; 60Hz and 40Hz for the OTS and EMTS systems respectively.

### Technical assessment

In the technical assessment of the two systems, two characteristics have been measured – static and dynamic tracking errors and system latency, with tracking errors being exclusively based on the translational part of the EMTS sensors’ localization data and the 3D position of individual markers acquired with the OTS. Although various technical parameters of these two systems have been measured and reported elsewhere [23–26], tracking error and system latency have been independently measured here to provide reference data on the performance of the two systems before moving on to the clinical assessment (see Section Clinical assessment below).

#### Tracking errors – static and dynamic measurements

**Fig. 1:**
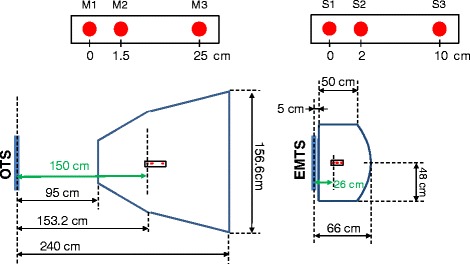
Measurement volumes of OTS (‘pyramid’) and EMTS (‘dome’) systems. The three *red dots* indicate the respectively the position of reference markers (M) and sensors (S) used for the measurement of static tracking errors

The tracking error in static measurements has been assessed by quantifying the spatial jitter of both systems, essentially the noise on the measured position of a stationary point. Three spaced points were localized to investigate the position dependency of spatial jitter. The measurement setup takes into account the different working volumes of the two systems, locating the three landmarks at distances of 150 cm and 26 cm from the OTS device or EMTS antenna (see Fig. 1). These values are estimated simulating the clinical settings for the tracking of thoracic surrogates with a couch mounted solution.

Dynamic distortions on the other hand have been quantified as the variation of the measured distance between two fixed points on a rigid frame as this was moved within the field of view of the system. The free hand motion of the frame covered the entire working volume of both systems at reasonably low speed (<20 cm/sec). For this purpose, we used the OTS ‘Rigid Body tool’ (Part Number 8700339) featuring two markers separated by 55 mm, and the EMTS ‘Standard Probe’ (Part Number 610065) which has two embedded sensors. For the latter probe, as the distance between these sensors is not provided by the manufacturer, this has to be estimated based on the average measurement resulting from a 2 secon﻿d acquisition in a controlled environment. From this, the distance between the two sensors could be determined to be 97 mm. The same testing protocol was applied for the small and large measurement volumes allowed by each of the systems, named ‘pyramid’ or ‘extended pyramid’ for the OTS, and ‘cube’ or ‘dome’ for the EMTS. The names reflect the shape of the field of view and are detailed in the respective product manuals (see Fig. 1 and [27]).

#### System latency

**Fig. 2:**
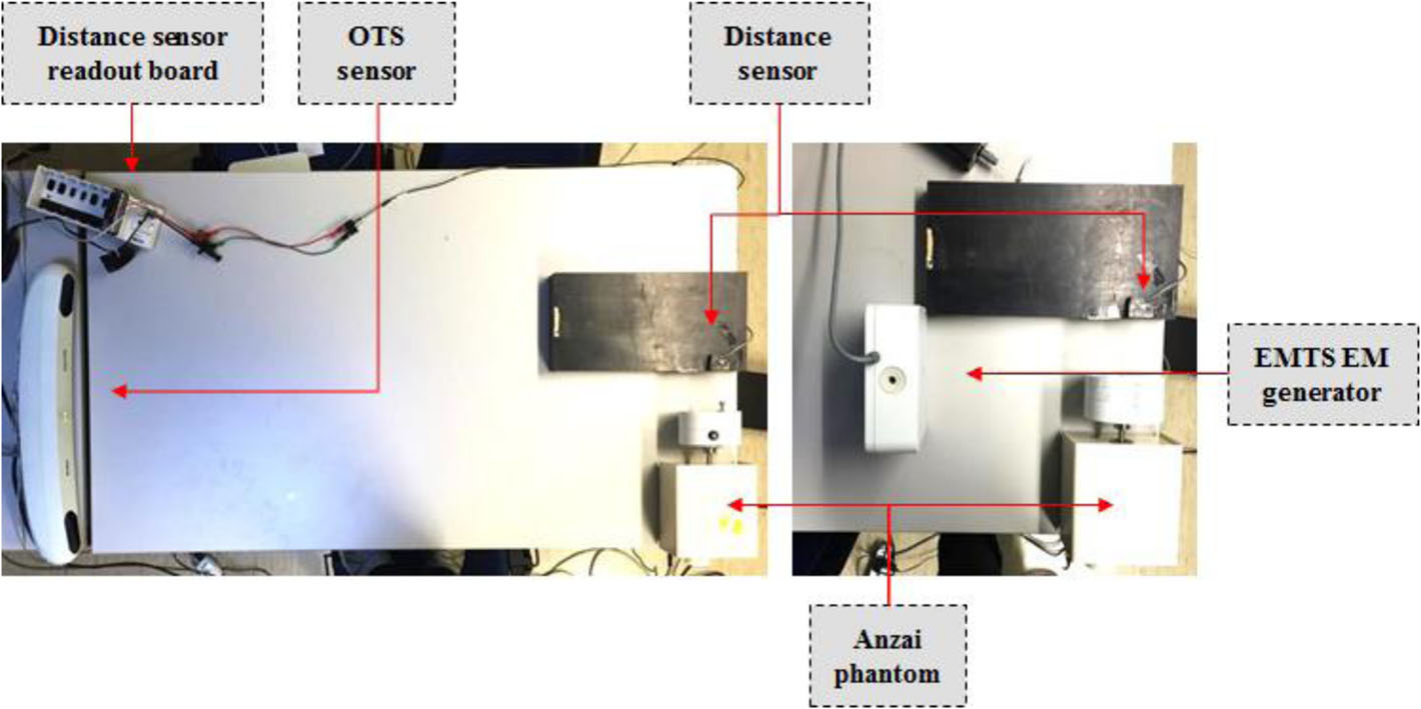
Setup for latency measurement with OTS (*left panel*) and EMTS (*right panel*). In the OTS setup, two infrared markers are visible on the Anzai phantom head. The catheter sensor (Part Number 610060) is used for the EMTS measurements

The Anzai respiratory phantom provided with Anzai Gating System AZ-733 V (Anzai Medical Co., Ltd, Tokyo, Japan) has been used to quantify system latency based on realistic scenarios of breathing motion (Fig. 2). The motion of the phantom has been measured concurrently by both tracking systems and an independent LED-based photoelectric distance sensor, which provides a reference measurement at 1 kHz (FADK 14U44790/IO, Baumer Electric AG, Frauenfeld, CH). The tracking systems and the readout board for the distance sensor (NI-9201 mounted in cDAQ-9172, National Instruments Corp., Austin, US) were connected to independent USB controllers on the host computer, and motion signals were logged by a dedicated software application that relies on the Windows API QueryPerformanceCounter (QPC) for the measurement of time intervals. The experimental data series were then smoothed (cubic Savitzky-Golay filter, zero phase [28]) and resampled with cubic spline interpolation using the time grid of the reference signal. The time delay was then estimated as the phase difference of the Fourier transform of the test and distance signals at the fundamental frequency of the phantom motion (0.25 Hz). In addition, we have verified the stability of the sampling period, by measuring the time interval between consecutive frames during an extended acquisition of 10 min.

### Clinical assessment

#### Practicality of tracking

**Fig. 3:**
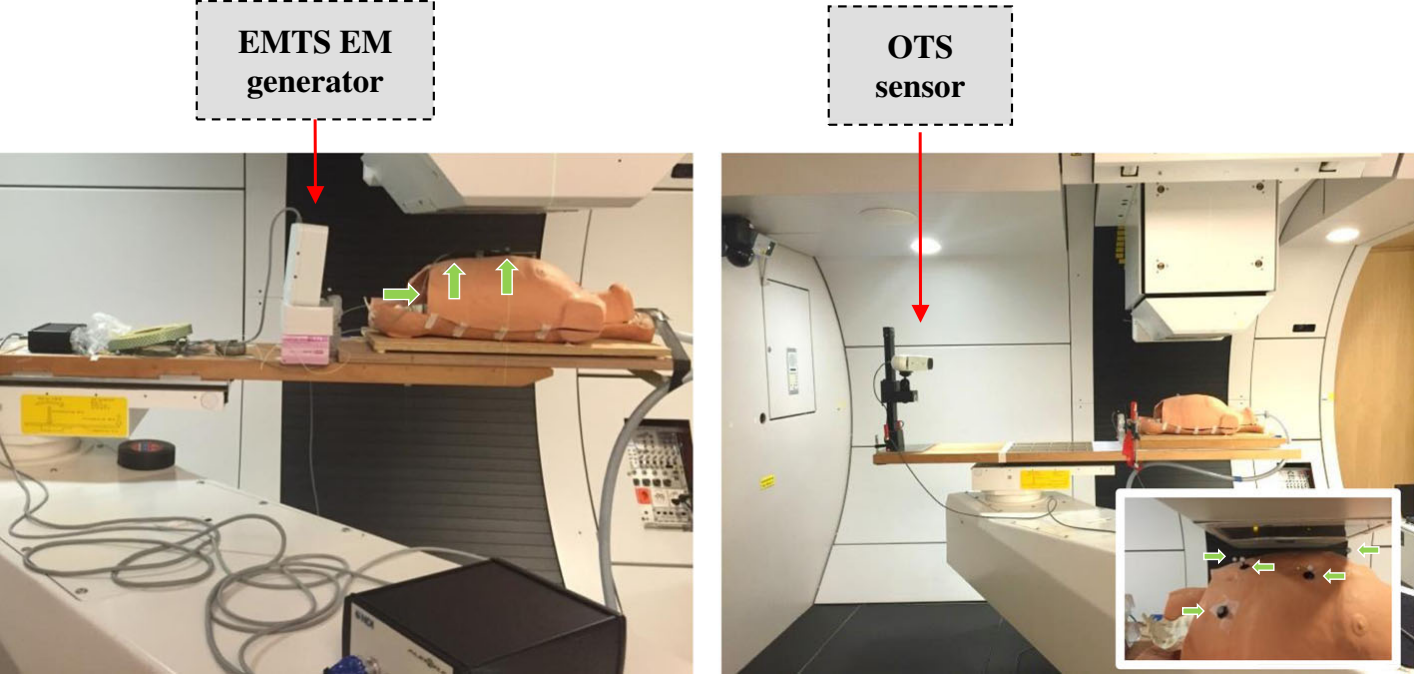
Testing of tracking system technologies in the Gantry 2 facility at PSI (couch in treatment position), the arrows point to 3 EM sensors (*left panel*) and 5 optical markers (*right panel*). The detail view in *right panel* shows the gantry configuration VI from Table 1

The practical aspects of integrating both systems into a PBS proton therapy facility have been addressed in the Gantry 2 proton treatment room [29] at the Paul Scherrer Institute (PSI). The availability of an in-room CT-on-rails (Siemens Somatom Sensation Open, Erlangen, D), together with the telescopic treatment nozzle of this gantry, enabled the investigation of two potential causes of tracking failure for optical and electromagnetic technologies respectively, i.e., marker visibility and magnetic field distortions. In addition, an anthropomorphic breathing phantom of a human thorax [30] has been used to mimic a realistic patient geometry in combination with three configurations of gantry angles and nozzle extraction values (Table 1 – I, II, III). These values are representative of clinical settings in PBS proton therapy, with the beam nozzle as close as possible to the patient to minimize the effect of air-gaps on the patient dose distribution. A configuration of 5 optical markers has been used, with three abdominal landmarks, one on the breastbone and a fifth on the upper chest, while two EM sensors were placed on the upper and a third on the lower abdomen (Fig. 3). Due to the asymmetric configuration of optical surrogates (Fig. 3, right panel), the OTS was tested in the presence of three additional conditions (Table 1 – IV, V, VI) designed to occlude the view of the most apical chest marker. As such, worst-case scenarios have been considered to identify possible limitations on the applicable treatment field angles.

**Table 1:** list of gantry angle and nozzle extraction configurations

Geometry	Gantry angle	Nozzle extraction	Nozzle-patient distance
I	0°	10 cm	~9 cm
II	−30°	10 cm	~7 cm
III	−30°	15 cm	~1.5 cm
IV	−30°	13 cm	~3.5 cm
V	30°	15 cm	~6.5 cm
VI	30°	20 cm	~2 cm

Beside the listed configurations of treatment settings, independent tests were carried out to quantify the effect of CT gantry position, rotation and X-ray exposure on the EM and optical localization of sensors. In particular, we have specifically considered two imaging protocols that are applied in the clinical routine at our institution, i.e., topograms (35 mAs, kVp: 120) and 3D CT images (200 mAs quality reference, kVp: 120, 1 sec gantry rotation time).

As would be used clinically, the OTS was installed at the foot end of the treatment couch whereas the EMTS field generator, due to the limited size of the measurement volume, was placed about 20 cm from the thorax phantom (see Fig. 3).

#### Motion tracking for 4DCT reconstruction

Finally, both systems were used to acquire motion signals for 4DCT reconstruction using the Siemens Open Interface, with the reconstructed images being compared with those obtained using the standard 4D imaging setup, i.e. the Anzai gating system AZ-733 V with a high sensitivity load cell sensor. CT images of the Anzai breathing phantom were acquired using 120 kV, 400 mAs quality reference, 2 mm slice thickness, 0.5 sec gantry rotation time and 0.1 pitch. The phantom was configured to execute the pre-set ‘quasi-respiratory’ motion curve featuring 15 cycles per minute with a nominal motion stroke equal to 20 mm. Amplitude-based image sorting was applied to define 10 motion phases for inhale and exhale phases at 0% 20% 45% 75% and 100% amplitude of the observed respiratory range of motion.

4DCT reconstructions were then analysed by comparing the observed phantom range-of-motion and temporal location of reconstructed phase points. For this purpose, an Acrylic sphere embedded in the moving part of the phantom was localized on each reconstructed phase by the use of a semi-automatic region growing segmentation. The centre of mass of the resulting segmented surface was then considered to define the sphere position, thus obtaining the phantom motion trajectory.

## Results

### Technical assessment

#### Tracking errors

**Fig. 4:**
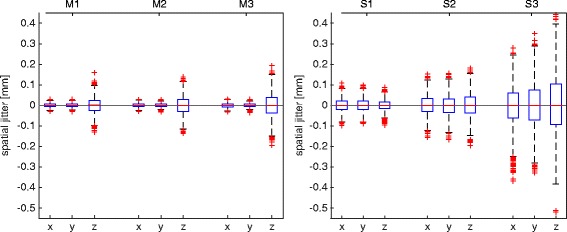
Spatial jitter for each coordinate of three markers and sensors measured respectively with OTS (*left panel*) and EMTS (*right panel*)

The quantification of spatial jitter was based on the localization of three stable points identified by individual infrared markers, a catheter sensor and a multi-sensor stylus probe located at different distances from the tracking systems (see Fig. 1 for measurement setup). The measurement distance with respect to the position sensor does not affect the measurement precision for OTS, but does for the EMTS, where EM field decay influences the quality of tracking as shown in Fig. 4. Root Mean Square (RMS) errors for markers tracked by the OTS system were always below 0.06 mm over 60 sec of acquisition, whereas for the EMTS, this increased from 0.05 mm to 0.2 mm as a function of distance from the measurement antenna.

**Fig. 5:**
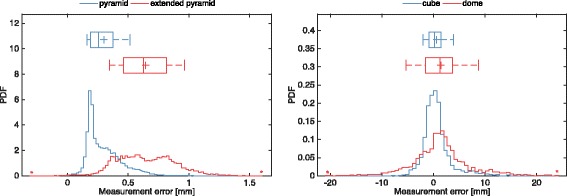
Probability density function of dynamic distortion in optical (*left panel*) and electromagnetic tracking (*right panel*) when using large (*red*) and small (*blue*) measurement volumes

As described above, dynamic distortion was quantified by the RMS error in the measurement of a distance between two points in motion. Measured distortions for the OTS were 0.33 mm and 0.69 mm respectively for the ‘pyramid’ and the ‘extended pyramid’ calibration volumes. Larger discrepancies were measured for the EMTS, with 2.83 mm and 5.73 mm RMS errors being measured using the ‘cube’ and the ‘dome’ volumes. A more detailed analysis of the error distributions shows how maximal errors in the optical measurements are rare and mostly located at the border of the calibration volume. Indeed, the interquartile ranges (IQR, 75^th^-25^th^ percentiles) of errors for the two measurement volumes were respectively 0.18 mm and 0.35 mm (Fig. 5, left panel). Conversely, large tracking errors during EM tracking were more frequent and even located in the central region of the measurement space, increasing the error IQR up to 2.33 mm and 5.18 mm respectively for small and large measurement volumes (Fig. 5, right panel).

#### System latency

The system latencies, assessed in three subsequent measurements lasting 1 min each, were determined to be 16.6 ± 1 msec and 31.6 ± 1 msec (mean ± std.dev.) for the OTS and EMTS systems, respectively. In addition, stable sampling periods were maintained during 10 min of tracking. The OTS sampling period matched the nominal values (f = 60Hz, T = 16.6 msec) with a narrow standard deviation: 16.65 ± 0.5 msec. Similarly, we measured deviations of 24.99 ± 2.7 msec for the EMTS system, working at 40 Hz (T = 25 msec). No frames were lost during any of the acquisitions.

### Clinical assessment

#### Practicality of tracking

The CT scanner considered in our study, featuring a 80 cm gantry bore, did not interfere with visibility of the markers on the thorax phantom for the OTS system, as this could be mounted on the treatment table. However, on the Gantry, it is always possible that views of the upper chest landmarks are occluded for certain combinations of gantry angle and nozzle position, although the abdominal and breastbone markers could be tracked in all tested geometries (see Table 1). This is largely dependent on the beam nozzle design and the height of the camera sensor with respect to the patient surface. In our tests, the OTS was placed 50 cm above the couch top and for nozzle-patient distances below 2 cm (Table 1 – configuration VI), the apical marker was completely occluded from the camera view. When clear line-of-sight is provided however, optical tracking is not affected by the measurement environment and allows the 3D localization of markers with the same accuracy as quantified in laboratory conditions (see Section Results - Technical assessment - Tracking errors – Fig. 4).

The testing of the EMTS started with the assessment of the tracking error on three sensors (two of them rigidly fixed on the stylus probe) that could result from the interference due to motion of the CT gantry (without X-ray exposure). The CT scanner was moved to two positions, approximately 35 mm and 25 mm away from the EM antenna. The transition from one position to the other resulted in a 3D measurement error of up to 7.3 mm for the most off-axis sensor, and up to 6 mm mismatch in the distance between the fixed sensors. In addition, the CT gantry rotation induced additional periodic oscillations on the measurements, resulting in up to 17 mm and 25 mm (75^th^ percentile) apparent displacements of the sensors in the two CT positions respectively. This effect is particularly critical when the CT is located very close to the EM antenna, about 25 cm in our setup. In this case, tracking failures are likely to occur, resulting in periodic gaps of missing data in the acquired trajectories.

**Fig. 6:**
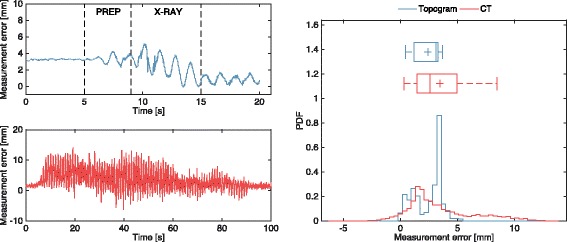
Measurement distortion during EM tracking in close proximity of a CT scanner. Topogram (*top-left panel*) divided in CT preparation (PREP) and acquisition (X-RAY) stages, and 3D CT imaging (*bottom-left panel*) acquisition protocols. Probability density function of measurement error is shown in the *right hand panel*

These environmental distortions are clearly visible when EM is used during topogram and CT imaging as shown in Fig. 6, where the tracking error in the relative distance measured between fixed sensors is reported as a function of the acquisition time. The baseline of tracking errors decreases proportionally with the position of the CT gantry that moves away from the EM antenna during the acquisition. Nevertheless, significantly large tracking errors are reported for both topograms and CT, up to 4.27 mm and 10.53 mm (97.5th percentile) respectively. In addition, large oscillations started during the preparation phase of the CT acquisition protocol (Fig. 6, top-left panel), attributable to the activation of the X-ray tube, and substantial detrimental effects were also observed due to CT gantry rotation, X-ray source activation and CT-motion during imaging (Fig. 6, bottom-left panel).

Finally, we investigated the effect of treatment gantry position and nozzle extraction on EM tracking. Relative displacements of a given configuration of three sensors of up to 29.3 mm and 22.9 mm were measured respectively after a 30° gantry rotation (Table 1, I → II configurations) and 5 cm nozzle extraction rotation (Table 1, II → III configurations). Order of magnitude errors comparable to those of the distance measurements when considering the two fixed markers on the stylus probe, were thus observed.

#### 4D CT images reconstruction

The environmental factors affecting the EM system outlined above precluded the use of the EMTS for the monitoring of respiratory motion during 4DCT acquisitions. Thus, only the OTS was benchmarked against the Anzai gating system connected to the high sensitivity load cell sensor. Before proceeding to 4DCT image reconstruction, the optical motion signal was first processed to compensate for the measurement latency (16.6 msec, refer to Section Results - Technical assessment - System latency) and smoothed to ease the detection of inhale and exhale points in the Siemens Syngo reconstruction platform.

The motion of the Anzai phantom, measured with the optical tracking system, was compared with its trajectory segmented on the 4DCT images reconstructed using either OTS or Anzai based motion trajectories. Principal component analysis was applied to identify the first component of motion of the 4DCT-derived and optical motion trajectories, thus obtaining comparable mono-dimensional signals even though the optical tracking system and the CT scanner were not geometrically calibrated. Regarding the measured range of motion, a small discrepancy was observed between the optical measurements (19.82 mm) and the OTS-4DCT and Anzai-4DCT reconstructions, equal to 19.32 mm and 19.29 mm respectively.

**Fig. 7:**
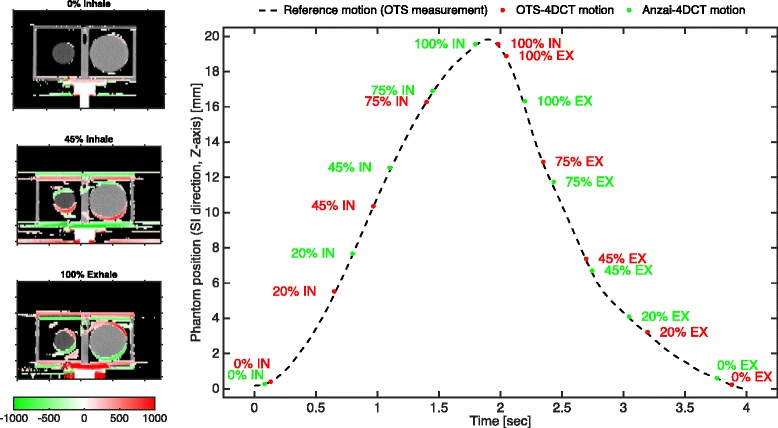
reconstructed phantom motion from 4DCT images sorted by using amplitude-based binning criteria with optical (OTS-4DCT) and Anzai (Anzai-4DCT) motion data. Coronal image cuts are shown in the *left panel* for the two worst cases (45% Inhale – 100% Exhale) using the *red*-to-*green color* map to show the difference of pixel intensities [Hounsfield Units - HU] in OTS-4DCT with respect to the Anzai-4DCT. The best case “0% inhale” is shown for comparison. In the *right panel* the motion trajectories derived from the two 4DCT dataset are compared with the nominal phantom position in the entire breathing cycle

In order to compare the temporal location of reconstructed phase points in OTS-4DCT and Anzai-4DCT, a single breathing cycle was extracted from the optical measurement and considered as the nominal trajectory (dashed line in Fig. 7). The time instants belonging to the Anzai-reconstructed CT phases were then estimated by matching the segmented phantom position with this nominal motion. OTS-4DCT and Anzai-4DCT phases are overlaid for comparison in Fig. 7. During the 4D CT reconstruction process, the amplitude peaks in the motion signal are used to identify the sequence of inhale and exhale respiratory segments required by the binning algorithm. Systematic time shifts between the peaks detected by the CT software on the Anzai motion data, and those identified on the optical motion signal led to time mismatches between the reconstructed 4DCT phases. The impact on the reconstructed images however depends on the specific shape of the respiratory motion signal.

## Discussion

In this work, a comparative analysis of optical (OTS) and electromagnetic (EM) tracking systems for monitoring respiratory motion in image-guided proton therapy has been reported. This has covered both the technical and clinical aspects of integrating such tracking technologies into the treatment process for 4D treatments on a PBS proton gantry.

A wide range of solutions for non-invasive monitoring are currently available, such as the Varian Real-time Position Management (RPM, Varian Medical Systems Inc., Salt Lake City, US) and the Anzai Respiratory Gating System (AZ-733 V, Anzai Medical Co., Ltd, Tokyo, Japan) to name the two most popular systems used for radiotherapy [31, 32]. Despite the use of calibration procedures however, it is often difficult to relate the load variation of the Anzai belt, or the laser sensor measurement, to the geometrical patient displacement in anatomical coordinates, making these solutions best suited for the detection of intra-fraction relative motions. Although the RPM in some configurations provides 3D positional information, this is limited to a single anatomical point where the six-dot marker block is located. An alternative approach consists of using tracking technologies to localize the whole patient surface, or multiple landmark points distributed on it, thus increasing the amount of information available for day-to-day verification of motion reproducibility and modelling. For this purpose, optical solutions are largely adopted in photon radiotherapy and, when coupled with internal/external correlation models, can be used to achieve real time tumour tracking [33]. Such point-based measurements generally require simpler technology than full optical surface tracking, whose sophisticated processing algorithms limit the frame rate and lengthen latency. For this reason, in real-time applications such as gated imaging and treatment, they are generally preferred, with surface-based solutions being best suited for patient setup, where time requirements are less strict. Even the market leading technology for surface guided radiotherapy - VisionRT (VisionRT Ltd., UK) - falls back to tracking points within a small region of interest on the patient thorax when used for gating (GateCT module, [34]). As such, in this work, we have assessed a cost effective optical tracking system, which is compact enough to be mounted directly on the treatment table.

More recently, electromagnetic tracking has also been applied in radiotherapy to localize wireless internal transponders using the ‘Calypso GPS system for the body**®**’ system (Varian Medical Systems Inc., Salt Lake City, US) [35, 36]. However, the need to maintain a large antenna close to the patient complicates its integration in proton therapy, where all the materials present in the treatment field have to be accurately qualified. Moreover, alternative commercial solutions that enable the electromagnetic localization of wired sensors are also available and come with reduced price with respect to Calypso**®** (Varian Medical Systems Inc., Salt Lake City, US). Even though their use is mostly documented for computer assisted procedures such as minimally invasive surgery [23] and interventional radiology settings [24], wire-based EM solutions represent a valid alternative to optical systems for real time monitoring of external patient motion, and this has been the EM solution adopted here.

The tracking of respiratory motion does not imply strict requirements on absolute accuracy of localization, but static and dynamic distortions of relative motions, as well as spatial jitter, could affect the quality of the acquired motion information. As such, tracking precision for the two systems under evaluation has been first tested in a controlled laboratory environment. In agreement with Franz et al. [23] and Khadem et al. [25], both technologies allow for the localization of static fiducials with sub-millimetre jitter. However, we have found significant dynamic and environmental distortions with the EMTS measurements of up to a few millimetres, which would significantly limit the potential of the EM approach for the tracking of respiratory motion in a clinical environment for gantry based, image-guided proton therapy.

On the system latency tests however, both systems performed well, with measured delays lower than those reported by the manufacturer. Even though the readout latency of the benchmark signal biases the estimation of absolute delays, it does not affect the differential analysis of measured latencies. The 16 msec delay measured between acquired electromagnetic and optical data series shows a very similar performance of the two systems under testing. Overall, the low motion speed, typical of respiratory signals, has certainly a positive effect on the measurement performance, as well as the small number of tracked surrogates in both cases, which reduces the computational workload of the systems. Finally, the high stability of the sampling rate is expected to reduce distortions in the reconstructed motions [37].

Optical surface tracking has been found to be robust against environmental factors, with no appreciable tracking errors due to CT imaging or changes in the PBS gantry position; however, the line-of-sight requirement implies constraints on the treatment geometry. Nevertheless, the simulation of a representative set of treatment geometries as applied in clinical routine (see Table 1), tested using a realistic thorax phantom to mimic patient anatomy, confirmed the effective tracking of abdominal and breastbone landmarks in even worst-case conditions where the treatment nozzle is in close proximity to the patient surface (Table 1 - configuration VI). Even though in our tests the position sensor was kept parallel to the couch table top, additional flexibility in setup options such as variable height and orientation could likely overcome most of the visibility issues occurring with particular gantry angles and nozzle extraction values. On the other hand, the application of electromagnetic tracking, albeit overcoming the line-of-sight restriction, is critically limited by environmental disturbances. The substantial decrease measured in the tracking accuracy for the EM system during imaging is in agreement with the results of Maier-Hein et al. [24] and Yaniv et al. [26], who report errors of a few millimetres in relative localization of sensors in an interventional radiology setting.

We have identified three main sources of distortion contributing to this drop in EM tracking accuracy during imaging: (i) variation in the distance between the CT scanner and the magnetic field generator, (ii) CT gantry rotation and (iii) X-ray source activation. Similarly, the presence of the treatment nozzle within the measurement workspace affects localization accuracy. Moreover, the positional shift of localized sensors due to the motion of the treatment gantry could reach up to a few centimetres, hampering the use of such EM technology for accurate motion monitoring during treatment. The complete calibration of environmental factors affecting the measurement is however non-trivial due to the complex superimposition of effects in a real-case scenario, where patient specific configuration of sensors, imaging protocols and treatment settings are applied.

Finally, we have compared 4DCT reconstructions obtained using the optical tracking system and the Anzai gating system. The comparison is based on the Anzai respiratory phantom in ‘quasi-respiratory’ periodical motion. The difference in the measured motion range between the two reconstructions and the predefined trajectory was below 0.5 mm. This is reasonable considering the cranio-caudal direction of the motion, where the CT images have a 2 mm slice thickness. The simple breathing phantom used in this study ensured the constant phase matching between the motion signals captured by the two monitoring systems. However, a significant mismatch in the temporal location of the reconstructed motion phases is observed even though the same binning scheme is applied. The inaccurate inhale and exhale point detection, combined with the steep motion gradients, lead to a significantly shifted 100% exhale phase and non-symmetric 100% inhale and 100% exhale phases in the Anzai-based reconstruction. The latency between Anzai and OTS measurements does not completely explain this misbehaviour. Indeed, the signal noise, as well as undocumented technical details about the detection of inhale and exhale peaks for the AZ733V [37], are the primary causes of these phases ‘shifts’ between the systems. Moreover, the different physical quantities measured by the two systems (displacement and pressure load variation respectively), together with the unknown relation between the phantom head motion and pressure applied on the load transducer, are further sources of uncertainty. This comparison of 4D CT reconstructions highlights the critical dependency between the applied motion monitoring system and the time location of the reconstructed motion phases [32, 37]. Thus we conclude that, for PBS proton therapy purposes, the same monitoring system should be applied for the imaging and treatment to ensure consistency of detected phases.

## Conclusions

Optical and electro-magnetic tracking systems were compared for respiratory motion monitoring in gantry based, image-guided proton therapy. Even though the need for a clear line of sight for optical localization sets constrains the configuration of the surface fiducials, this does not imply significant geometrical limitations on the treatment geometry. Conversely, reduced accuracy hampers the straightforward application of electromagnetic solutions during CT imaging, commonly applied in the current radiotherapy clinical practice. Moreover, limited accuracy in dynamic tracking implies additional efforts to process the acquired motion traces acquired with the electromagnetic system. In the perspective of tracking technology for integration into a PBS proton therapy environment, at least at the current stage of technology, the optical solution offers the best potential, being the most robust against environmental factors, whilst also providing the highest spatial accuracy.
